# Intraocular pressure and ocular biomechanical parameters vary between generations of the ocular response analyzer in healthy and ectatic eyes

**DOI:** 10.3389/fmed.2025.1605641

**Published:** 2025-07-24

**Authors:** Phillip T. Yuhas, Maddison M. Fortman, Michael Nye, Ashraf M. Mahmoud, Cynthia J. Roberts

**Affiliations:** ^1^College of Optometry, The Ohio State University, Columbus, OH, United States; ^2^Department of Ophthalmology and Visual Sciences, College of Medicine, The Ohio State University Wexner Medical Center, Columbus, OH, United States; ^3^Department of Biomedical Engineering, College of Engineering, The Ohio State University, Columbus, OH, United States

**Keywords:** intraocular pressure, corneal hysteresis, waveform parameters, tonometry, keratoconus, cornea

## Abstract

**Introduction:**

This study evaluated the agreement between a third-generation (G3) ocular response analyzer (ORA) and a first-generation (G1) ORA, and tested the ability of the keratoconus match index (KMI) to identify keratoconus.

**Methods:**

Healthy participants (*n* = 149 eyes) and participants with keratoconus (*n* = 78 eyes) were enrolled for this study. Four measurements were taken bilaterally using the G1 and G3 ORA. Goldmann-correlated intraocular pressure (IOPg), corneal-compensated IOP (IOPcc), corneal hysteresis (CH), waveform score, KMI, and waveform parameters area under the first applanation peak (p1area), area under the second applanation peak (p2area), width of the first applanation peak (w1), width of the second applanation peak (w2), height of the first applanation peak (h1), and height of the second applanation peak (h2) were recorded from the measurement with the highest waveform score in the left eye. Paired *t*-tests or Wilcoxon signed-rank tests were used to assess agreement between the devices, and receiver-operating characteristic curves determined the ability of KMI to identify eyes with keratoconus.

**Results:**

There was no difference in IOPcc or IOPg between the devices in both cohorts. CH was significantly greater for the G3 than for the G1 in healthy participants but not in keratoconus participants. For both cohorts, measurements of waveform score, KMI, p1area, p2area, w2, h1, and h2 were greater for the G3 than for the G1. Only w1 was smaller for the G3 than for the G1. There was no difference in the ability of KMI to differentiate ectatic from healthy eyes between the devices.

**Discussion:**

Although the G1 and G3 can identify keratoconus using KMI, there is meaningful variation between them in IOP and biomechanical outcome parameters. Thus, clinicians and researchers should compare results between the devices with caution and should state which generation produced the data.

## 1 Introduction

The ocular response analyzer (ORA; Reichert, Depew, NY, USA) is a dynamic bidirectional tonometer that uses infrared light reflected from the cornea during deformation by a puff of air to calculate two measures of intraocular pressure (IOP). Although Goldmann-correlated IOP (IOPg) is associated with IOP measured using Goldmann applanation tonometry (1), it can produce an underestimation of less than 1 mmHg (2). Corneal-compensated IOP (IOPcc) uses an empirically derived formula to mitigate the confounding effects of central corneal thickness and corneal stiffness on measured IOP (3). Unlike most other tonometers, the ORA produces additional measurements of the biomechanical parameters of the eye. Corneal hysteresis (CH) is a viscoelastic parameter that represents the ability of the eye to dissipate energy. Low CH is associated with the loss of structural and functional integrity in glaucoma (4, 5), and it predicts conversion from ocular hypertension to glaucoma (6). CH is also reduced in patients with keratoconus, compared to healthy controls, but its diagnostic potential is limited by substantial overlap between the two populations (7).

The ORA produces 38 additional metrics that describe the shape of its twin-peaked applanation waveform (8). These outcomes are not displayed on its clinical interface and must be downloaded from the device. Analysis of waveform parameters has emerging utility for detecting and monitoring ocular disease (9). Aoki and colleagues found that waveform parameters correlate significantly with the progression of visual field deterioration in glaucoma (10). The keratoconus match index (KMI) is a keratoconus-specific set of seven waveform parameters derived from a neural-network analysis of patients included in the internal databases of the device, to represent the agreement between the pressure-applanation waveforms of a given eye and the typical pressure-applanation waveforms of a keratoconic eye. Labris and colleagues used the KMI to differentiate patients with keratoconus, even subclinical disease, from healthy controls with accuracy greater than that with CH alone (11, 12). Thus, biomechanical assessment of the eye using the ORA has the potential to supplement corneal-imaging techniques for the detection of keratoconus.

The initial iteration of the ORA, the G1, first received approval from the United States Food and Drug Administration in 2004. In 2016, the manufacturer introduced a third generation of the device, the G3. The G3 ORA was a substantial departure from the first two generations of the device because its working distance was decreased from 11.3 mm to 8.3 mm and because improvements were made to its air-puff-delivery and applanation-detection systems. These changes attenuated turbulence in the air puff and reduced noise in the infrared signal to optimize repeatability (8). By failing to report which generation of the ORA was used for data collection, most researchers and clinicians assume that the G3 ORA is interchangeable with earlier generations of ORA. It is likely, however, that the aforementioned updates to the G3 ORA altered its pressure-applanation waveforms. Subsequent changes to the outcome metrics of the G3 ORA have not been evaluated against the G1 ORA. Moreover, KMI was not included in the software package of the G3 ORA; thus, its performance has not been tested on the updated device. The purposes of this study are to evaluate the agreement between the outcome metrics of G3 ORA and G1 ORA and to test the ability of KMI to identify patients with keratoconus on the updated device.

## 2 Materials and methods

This cross-sectional study conformed to the tenets of the Declaration of Helsinki. The Biomedical Institutional Review Board at The Ohio State University (OSU) approved the research (protocol number 2022H0067), and all participants provided written informed consent before enrollment. The study presented here is part of a larger investigation into ocular biomechanics.

### 2.1 Study participants

Adults (aged ≥18 years) were recruited for this study prospectively from the students, faculty, staff, and patient population at the OSU College of Optometry between May 2023 and March 2024. Enrollees were binned into one of two cohorts. The keratoconus cohort comprised participants with diagnosed keratoconus (ICD-10 codes H18.60, H18.61, or H18.62), as determined by an optometrist or ophthalmologist via interpretation of corneal topography and/or tomography, slit lamp biomicroscopy, and consideration of family history and environmental factors, such as eye rubbing. There are no universally accepted criteria for the diagnosis of keratoconus (13). Participants in the healthy cohort had clear and regular corneas. Exclusion criteria for both cohorts included glaucoma or other optic neuropathies, ocular hypertension, diabetes mellitus, a history of ocular surgery, including corneal cross-linking but excluding uncomplicated cataract extraction, and use of orthokeratology contact lenses.

### 2.2 Data collection

All participants sat for one identical study session. First, self-reported age, sex, and race or ethnicity were recorded, and contact lenses were removed, if applicable, shortly before data collection. Then, simulated corneal topography values were acquired from both eyes using a commercial device (E300; Medmont International, Nunawading, Austria). Next, four measurements were taken from both eyes using two generations of the ORA, namely G1 and G3. The order of measurements made from the two devices alternated between participants to avoid a potential order effect. Standard clinical outcomes from the ORA included IOPg, IOPcc, CH, and waveform score, a metric of signal strength. KMI was generated automatically on the G1 ORA but had to be calculated offline for the G3 ORA using waveform parameters and an algorithm provided by the manufacturer in Excel (Microsoft, Redmond, WA, USA). Additionally, waveform parameters p1area, p2area, w1, w2, h1, and h2, which have been described elsewhere (14), were downloaded from the devices as easily interpreted representations of the shape (i.e., height, width, and area) of the twin-peaked ORA applanation waveform.

### 2.3 Data analysis

ORA data from the measurement with the highest waveform score were analyzed (15). The left eye of healthy participants was considered to account for a small, but statistically significant, order effect occurring in repeated measurements made by the ORA on normal eyes (16). Known inter-eye differences in disease severity and biomechanical parameters allowed both eyes of the keratoconus participants to be considered (17–20). For both cohorts, only eyes that had measurements from both devices were included for final analysis.

The Shapiro–Wilk test assessed the normality of all outcomes. Then, five analyses tested variability and the agreement between the G1 ORA and G3 ORA. First, inter-device differences between means (parametric data) or medians (non-parametric data) were evaluated using two-tailed paired *t*-tests or Wilcoxon signed-rank tests, respectively. Agreement between the devices was quantified further using linear regression and Bland–Altman analyses with accompanying coefficients of repeatability (C_R_) (21, 22). Fourth, the population-based coefficient of variation (C_V_) was calculated for all ORA outcome measures. Finally, receiver-operating characteristic (ROC) curves were generated to test the ability of each device to parse healthy participants from keratoconus participants using KMI. The difference in area under the ROC curves was assessed using the Chi-squared test. For all outcomes, the Bonferroni correction for multiple comparisons was used to set the statistical-significance threshold at *α* < 0.001.

## 3 Results

The demographics of the keratoconus cohort were reported previously (14). Briefly, 50 participants with keratoconus (mean ± standard deviation age = 39 ± 14 years; 28% female) were enrolled. Of the total participants, 58% self-reported as White, 30% as Black, 6% as Hispanic, and 6% as mixed-race. Of these 100 eyes, measurements could be acquired on 80% of them using the G1 ORA and on 94% of them using the G3 ORA. Thus, a total of 78 eyes (*n* = 78) were analyzed in the keratoconus cohort. Simulated keratometry values were 47.5 ± 7.85 D in the flat meridian and 51.7 ± 9.81 D in the steep meridian.

A total of 153 participants were enrolled in the healthy cohort (age = 39 ± 17 years; 61% female). Of these, 82% self-reported as White, 5% as Black, 1% as Hispanic, 8% as Asian, and 2% as mixed-race. Race and ethnicity were unknown in 1% of the healthy participants. Both the G1 ORA and the G3 ORA were able to acquire measurements on 99% of the 153 left eyes. Thus, 149 eyes (*n* = 149) were analyzed in the healthy cohort. Simulated keratometry values were 43.1 ± 1.51 D in the flat meridian and 44.2 ± 1.45 D in the steep meridian.

### 3.1 Inter-device agreement

Tables 1, 2 contain the parametric and non-parametric inter-device differences, respectively, for the healthy participants. There were no statistically significant differences between the G1 ORA and G3 ORA for IOPg and IOPcc. There were statistically significant differences between the devices for all other outcome measures. The values of h1, CH, waveform score, KMI, p1area, p2area, w2, and h2 were all larger in the G3 ORA than in the G1 ORA. Only w1 was larger in the G1 ORA than in the G3 ORA. The keratoconus cohort produced similar results, which are represented in Table 3 (parametric data) and Table 4 (non-parametric data). There were no statistically significant differences between the devices for IOPg and IOPcc, but h1, CH, waveform score, KMI, p1area, p2area, w2, and h2 were all larger in the G3 ORA than in the G1 ORA. Again, only w1 was larger in the G1 ORA than in the G3 ORA.

**Table 1:** Differences in parametric outcome parameters between the G1 ocular response analyzer (ORA) and the G3 ORA in the left eye of healthy participants (*n* = 149).

Outcome metric	G1	G3	*p*-value
IOPg (mmHg)	14.2 (3.15)	14.5 (3.06)	0.07
IOPcc (mmHg)	14.4 (3.38)	14.1 (3.00)	0.14
h1	461 (70.5)	527 (77.5)	<0.001*

Values are means (one standard deviation). *Statistically significant according to paired *t*-test with Bonferroni correction. IOPg, Goldmann-correlated intraocular pressure; IOPcc, corneal-compensated intraocular pressure.

**Table 2:** Differences in non-parametric outcome parameters between the G1 ocular response analyzer (ORA) and the G3 ORA in the left eye of healthy participants (*n* = 149).

Outcome metric	G1	G3	*p*-value
Corneal hysteresis (mmHg)	10.7 (9.40, 12.1)	11.2 (10.3, 12.3)	<0.001*
Waveform score	7.83 (6.86, 8.41)	8.18 (7.58, 8.87)	<0.001*
Keratoconus match index	1.01 (0.80, 1.21)	1.94 (1.69, 2.14)	<0.001*
p1area	4,449 (3,928, 5,238)	5,027 (4,409, 5,574)	<0.001*
p2area	3,111 (2,705, 3,608)	6,251 (5,504, 7,150)	<0.001*
w1	24.0 (22.0, 25.0)	20.0 (19.0, 21.0)	<0.001*
w2	20.0 (18.0, 23.0)	28 (25.0, 30.0)	<0.001*
h2	339 (342, 442)	518 (473, 566)	<0.001*

Values are medians (interquartile range). *Statistically significant according to the Wilcoxon signed-rank test with Bonferroni correction.

**Table 3:** Differences in parametric outcome parameters between the G1 ocular response analyzer (ORA) and the G3 ORA in eyes with keratoconus (*n* = 78).

Outcome metric	G1	G3	*p*-value
Corneal hysteresis (mmHg)	8.59 (1.63)	8.98 (1.85)	<0.001*
Keratoconus match index	0.38 (0.46)	0.86 (0.80)	<0.001*
p1area	2,979 (1191)	3,209 (1650)	<0.001*
p2area	2032 (878)	3,456 (1860)	<0.001*
w2	18.8 (6.17)	25.5 (6.58)	<0.001*
h2	288 (112)	334 (166)	<0.001*
h1	461 (70.5)	527 (77.5)	<0.001*

Values are means (one standard deviation). *Statistically significant according to paired *t*-test with Bonferroni correction.

**Table 4:** Differences in non-parametric outcome parameters between the G1 ocular response analyzer (ORA) and the G3 ORA in eyes with keratoconus (*n* = 78).

Outcome metric	G1	G3	*p*-value
IOPg (mmHg)	9.10 (6.18, 11.9)	9.55 (6.85, 12.3)	0.003
IOPcc (mmHg)	12.2 (10.5, 14.1)	12.2 (10.7, 14.1)	0.55
Waveform score	6.13 (3.99, 7.22)	6.44 (4.74, 7.96)	<0.001*
w1	23.0 (21.0, 25.0)	19.0 (17.0, 21.0)	<0.001*
h1	323 (259, 407)	383 (245, 528)	<0.001*

Values are medians (interquartile range). *Statistically significant according to the Wilcoxon signed-rank test with Bonferroni correction. IOPg, Goldmann-correlated intraocular pressure; IOPcc, corneal-compensated intraocular pressure.

**Figure 1:**
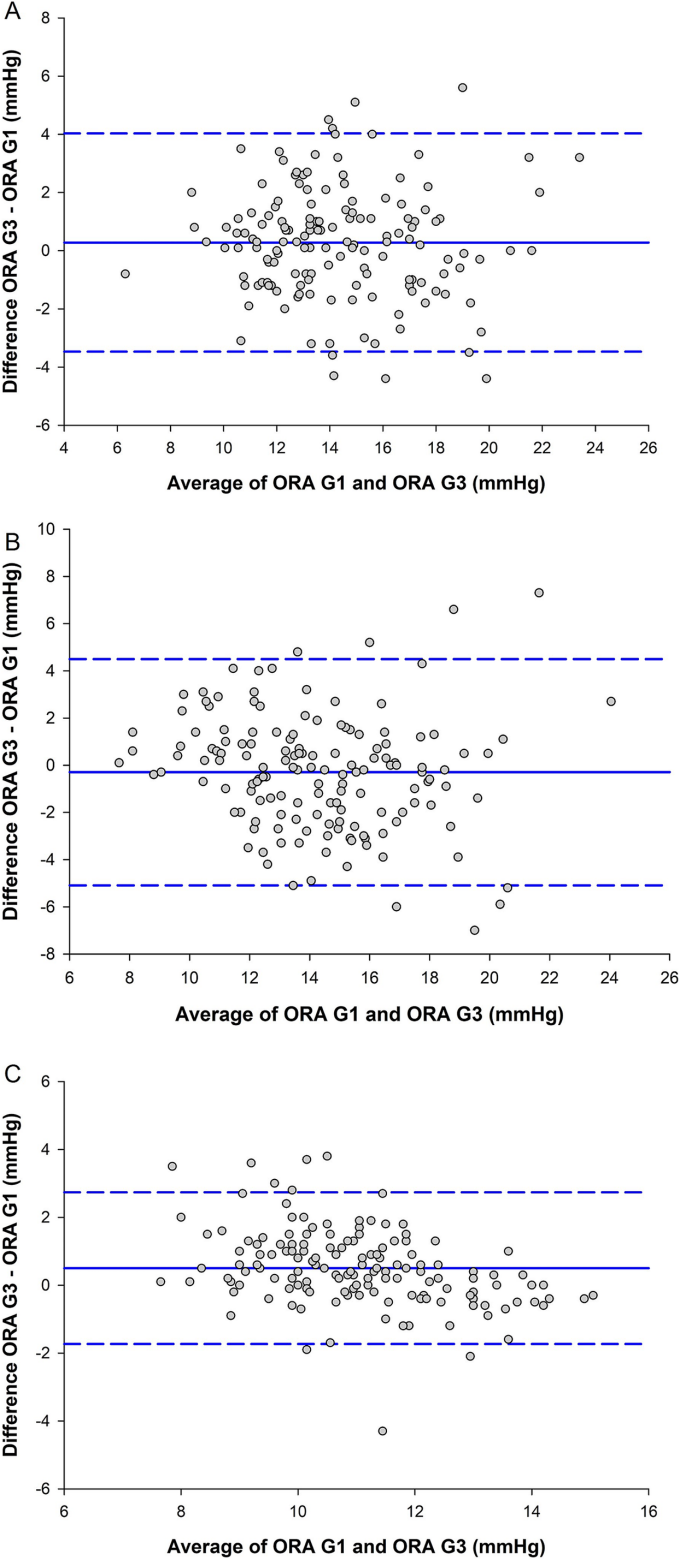
Agreement between the ocular response analyzer (ORA) G1 and ORA G3 for **(A)** Goldmann-correlated intraocular pressure (IOPg), **(B)** corneal-compensated IOP (IOPcc), and **(C)** corneal hysteresis (CH) in the healthy cohort (*n* = 149). The center solid blue lines are inter-device mean difference, and the flanking dashed blue lines are the limits of agreement, from 1.96 to −1.96 standard deviations.

Figure 1 contains Bland–Altman plots for IOPcc, IOPg, and CH in the healthy cohort. Bias toward one device over the other was low for both measurements of intraocular pressure, and there were strong-to-moderate and statistically significant associations between the devices for IOPg (*r* = 0.81, *p* < 0.001) and IOPcc (*r* = 0.71, *p* < 0.001) on linear regression analysis. However, the C_R_ was 5.30 mmHg for IOPg and 6.78 mmHg for IOPcc, indicating a wide range of measurement agreement. Although CH was like IOPg and IOPcc in that bias was low and linear agreement was moderate between the devices (*r* = 0.79, *p* < 0.001), its C_R_ was narrower at 3.16 mmHg. For the other ORA outcome measures in the healthy cohort, there was a moderate and statistically significant association between the devices for p1area (*r* = 0.68, *p* < 0.001). There were moderate-to-weak and statistically significant associations between the devices for KMI (*r* = 0.53, *p* < 0.001), p2area (*r* = 0.44, *p* < 0.001), and w2 (*r* = 0.43, *p* < 0.001). There were weak but statistically significant associations between the devices for w1 (*r* = 0.37, *p* < 0.001) and h1 (*r* = 0.36, *p* < 0.001). Associations were not-statistically-significant between the devices for waveform score (*r* = 0.20, *p* = 0.01) and h2 (*r* = 0.26, *p* = 0.001). The Bland–Altman plots for waveform score (*C_R_* = 3.78), KMI (*C_R_* = 0.89), p1area (*C_R_* = 2,328), p2area (*C_R_* = 3,206), w1 (*C_R_* = 7.32), w2 (*C_R_* = 11.4), h1 (*C_R_* = 231), and h2 (*C_R_* = 251) can be found in Supplementary Figure S1.

**Figure 2:**
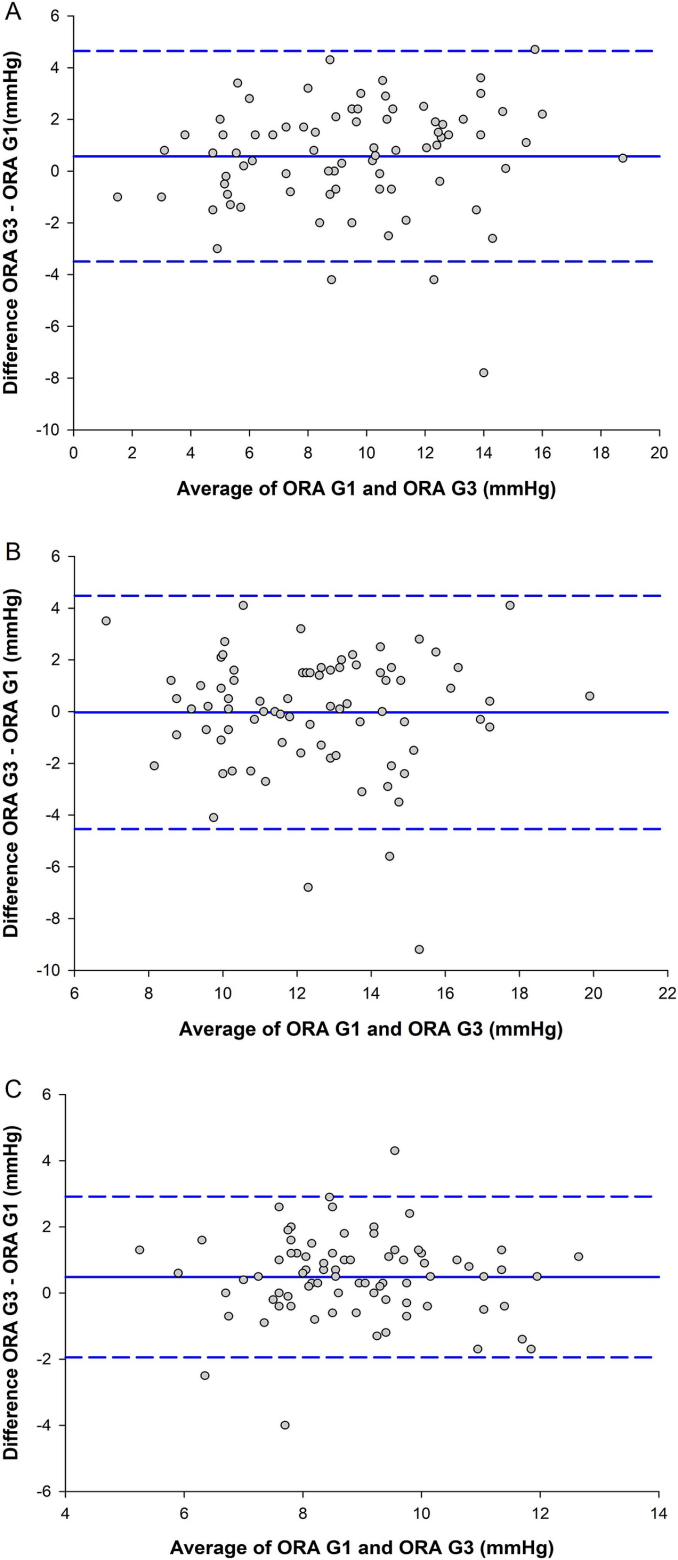
Agreement between the ocular response analyzer (ORA) G1 and ORA G3 for **(A)** Goldmann-correlated intraocular pressure (IOPg), **(B)** corneal-compensated IOP (IOPcc), and **(C)** corneal hysteresis (CH) in the keratoconus cohort (*n* = 78). The center solid blue lines are inter-device mean difference, and the flanking dashed blue lines are the limits of agreement, from 1.96 to −1.96 standard deviations.

Figure 2 contains Bland–Altman plots for IOPcc, IOPg, and CH in the keratoconus cohort. Similar to the results in the healthy cohort, there was minimal bias toward one device over the other for all three metrics, but C_R_ was 5.76 mmHg for IOPg, 6.37 mmHg for IOPcc, and 3.45 mmHg for CH. Linear associations between the devices were strong-to-moderate and statistically significant for IOPg (*r* = 0.85, *p* < 0.001) and CH (*r* = 0.70, *p* < 0.001), and were moderate and statistically significant for IOPcc (*r* = 0.66, *p* < 0.001). For the other ORA outcome metrics, there were strong-to-moderate and statistically significant linear associations between the devices for waveform score (*r* = 0.71, *p* < 0.001), KMI (*r* = 0.78, *p* < 0.001), p1area (*r* = 0.86, *p* < 0.001), h1 (*r* = 0.78, *p* < 0.001), and h2 (*r* = 0.79, *p* < 0.001). There were moderate-to-weak and statistically significant associations between the devices for p2area (*r* = 0.63, *p* < 0.001) and w2 (*r* = 0.46, *p* < 0.001). There was a weak but statistically significant relationship between the devices for w1 (*r* = 0.39, *p* < 0.001). The Bland–Altman plots for waveform score (*C_R_* = 4.65), KMI (*C_R_* = 1.12), p1area (*C_R_* = 2010), p2area (*C_R_* = 3,399), w1 (*C_R_* = 12.0), w2 (*C_R_* = 17.7), h1 (*C_R_* = 270), and h2 (*C_R_* = 234) can be found in Supplementary Figure S2.

### 3.2 Variation of measurements

Table 5 contains the C_V_ values for both devices in both cohorts. In general, C_V_ values trended higher in keratoconus participants than in healthy participants. In the healthy cohort, C_V_ trended slightly lower in the G3 ORA than in the G1 ORA, especially for the waveform parameters. This trend was reversed in the keratoconus cohort, where C_V_ trended larger in the G3 ORA than in the G1 ORA.

**Table 5:** Coefficients of variation for the G1 ocular response analyzer (ORA) outcomes and for G3 ORA outcomes in the healthy and keratoconus cohorts.

Outcome metric	Healthy participants (*n* = 149)		Keratoconus participants (*n* = 78)	
	G1 ORA	G3 ORA	G1 ORA	G3 ORA
IOPg	0.22	0.21	0.40	0.39
IOPcc	0.23	0.21	0.23	0.21
CH	0.17	0.13	0.19	0.21
WFS	0.16	0.12	0.40	0.40
KMI	0.32	0.18	1.2	0.94
p1area	0.24	0.19	0.40	0.51
p2area	0.28	0.19	0.43	0.54
w1	0.11	0.10	0.20	0.22
w2	0.20	0.13	0.33	0.26
h1	0.15	0.15	0.39	0.50
h2	0.20	0.14	0.39	0.50

IOPg, Goldmann-correlated intraocular pressure; IOPcc, corneal-compensated intraocular pressure; CH, corneal hysteresis; WFS, waveform score; KMI, keratoconus match index.

### 3.3 Identification of participants with keratoconus

**Figure 3:**
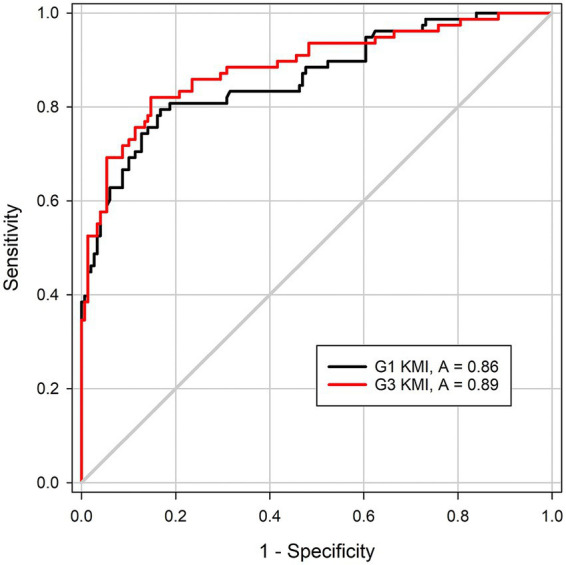
Receiver-operating characteristic (ROC) curves representing the ability of the keratoconus match index (KMI) to distinguish the keratoconus participants from the healthy participants using the G1 ocular response analyzer (ORA, black trace) and G3 ORA (red trace). A is area under the ROC curve.

Figure 3 contains the ROC curves that represent the ability of KMI to distinguish keratoconus participants from healthy participants using the G1 ORA and G3 ORA. The area under the ROC curve was not statistically significant between the devices (*p* = 0.35).

## 4 Discussion

To the knowledge of the authors, this is the first study to test the hypothesis that the reduced working distance and updated air-puff-delivery and optical-detection systems of the G3 ORA changed its outcome metrics, compared to the G1 ORA. In both cohorts, healthy and keratoconus, there were significant differences between the devices for all the biomechanical outcome metrics, CH, KMI, p1area, p2area, w1, w2, h1, and h2. CH, a viscoelastic measurement of energy dissipation, was higher in the G3 ORA than in the G1 ORA. In general, the peaks of the pressure-applanation waveforms were higher and wider in the G3 ORA than in the G1 ORA, resulting in greater areas under them in the former than in the latter. The exception to this result was w1, which was significantly wider in the G1 ORA than in the G3 ORA. The fact that the waveform score was significantly greater in the G3 ORA than in the G1 ORA in both cohorts suggests that the newer device achieves better alignment with the cornea than the older device. This interpretation is supported by the fact that the G1 ORA could acquire a measurement on only 80% of eyes with keratoconus, but the G3 ORA could acquire a measurement on 94% of them, which approaches the 99% capture rate on healthy eyes.

In aggregate, these results are strong evidence that the updates to the G3 ORA did change its outcome parameters, compared to earlier generations. These changes may have clinical and research significance. For example, although the median (healthy cohort) and mean (keratoconus cohort) differences between the devices for CH were small but statistically significant, the limits of agreement on Bland–Altman analysis were large, and the C_R_ values were above 3 mmHg in both cohorts. From other works, the average differences in CH between patients with glaucoma and healthy controls and between patients with keratoconus and healthy controls are typically less than 3 mmHg (7, 23, 24). Thus, changing between generations could mask clinically relevant differences in CH.

The interpretations of IOPg and IOPcc are similar to those of CH. There were no significant results between the G1 ORA and G3 ORA for both outcomes when differences in means and medians were assessed using the paired *t*-test (healthy participants) or the Wilcoxon signed-rank test (keratoconus participants), and there were strong-to-moderate linear associations between the devices. These realities are reflected in a bias near zero on their corresponding Bland–Altman plots. However, inspection of their limits of agreement and the accompanying C_R_ values suggests substantial variability between the devices, greater than 5 mmHg, even in the healthy cohort. Certainly, 5 mmHg is clinically meaningful when managing patients with open-angle glaucoma, in whom a change of 1 mmHg is associated with a 10% increase or decrease in risk for disease progression (25).

The clinical significance of the difference in waveform parameters between the devices is more difficult to establish than that of CH or IOP, as investigation into the diagnostic potential of the waveform parameters remains in its early stages. Based on the work that has been completed on the topic, it is known that relatively small increases in p2area are associated with worsening visual sensitivity in patients with glaucoma (10). Furthermore, Goebels and colleagues reported a difference in p2area between participants with keratoconus and controls, which falls within the range of variation between the G3 ORA and G1 ORA reported here (26). Additionally, the magnitudes of decreases in p1area, p2area, and h1 after laser refractive surgery reported by Landoulsi and colleagues fall within the limits of agreement of the two ORA generations (27). So too do changes in waveform parameters elicited by orthokeratology contact lenses in children (28). Thus, it is clear that the two devices cannot be used interchangeably. When possible, clinicians should note the generation of the device used and use the same generation device for serial measurements over time. Researchers should report on the generation of ORA used for data collection and should not extrapolate the results of an early-generation ORA to a G3 device.

Coefficient of variation (C_V_) was included as an additional metric of device performance. It is not surprising that C_V_ was higher in the keratoconus cohort than in the healthy cohort, regardless of the generation of the device. Irregularity in the curvature of the ocular surface in eyes with keratoconus likely elicits more scatter of the infrared photons used by the optical-detection system of the ORA than in healthy eyes. Moreover, as mentioned previously, deficits in the alignment of the G1 ORA with the keratoconic eye likely add to measurement variability. The differences in variability between the devices were substantially less than the differences between the devices within each cohort. There were modest trends of decreased variability in the G3 ORA in the healthy cohort and increased variability in the keratoconus cohort. The peaks of the applanation curve are blunted in keratoconus, especially central disease (14). Thus, it is not surprising that an ectatic cornea elicits more variability on the device that produces taller peaks, the G3, than on the one that generates shorter peaks. This study was not designed to determine whether the trend toward increased variability in the G3 waveform parameters hampers its diagnostic accuracy, so future work will have to address this issue. The C_V_ values for IOPg and IOPcc on the G1 ORA calculated in this study are nearly double those reported by Wang and colleagues (29). The reason for the discrepancy between the two studies lies in the way that C_V_ was calculated. Wang and colleagues calculated C_V_ for each parameter using the mean and standard deviation from three measurements on each participant. In this study, population-based C_V_ was calculated from the measurement with the highest waveform score for each parameter based on the mean and standard deviation of the entire study population. Since variability of a single measurement taken from many participants is larger than the variability of multiple measurements taken on one participant, the C_V_ values reported in this study cannot be compared directly to those from Wang and colleagues.

Even though their pressure-applanation waveforms differed, the two devices performed similarly when using KMI to differentiate healthy eyes from eyes with keratoconus, as represented by their areas under the ROC curves. The area under the ROC curve value in the G1 ORA lagged behind the values reported by Labiris and colleagues in frank ectasia and subclinical disease, 98 and 94%, respectively (11, 12). Taken together, these results suggest that KMI is due for an update. First, KMI needs to be optimized for the G3 ORA. Its robust pressure-applanation waveforms may yield more and different markers of ectasia than the waveforms of the G1 ORA, on which KMI was first developed. That is, the current iteration of KMI may be suppressing the full potential of the G3 ORA. Second, image analysis of the pressure-applanation waveforms of the ORA is likely well suited for machine learning applications to identify differences between healthy eyes and eyes with keratoconus. Such work is already being done on eyes with myopia (30), and multiple groups have applied machine learning to analyze images of keratoconus corneas generated by Cornea Visualization with Scheimpflug Technology (Corvis ST; OCULUS Optikgeräte GmbH, Wetzlar, Germany) (31, 32), an air-puff tonometer that uses a high-speed camera with Scheimpflug geometry to image the cornea during deformation. A next step in this line of research is to apply similar machine learning techniques to optimize the potential of the ORA, which may be more ubiquitous in primary eye clinics, where keratoconus is often first detected, than the Corvis ST, to detect early keratoconus in conjunction with traditional cornea imaging techniques.

This study has several limitations. First, the stage of keratoconus was not graded; thus, the agreement between the devices could not be assessed as a function of disease severity. Second, this study was cross-sectional in eyes with previously diagnosed keratoconus. The true value of metrics like KMI is to predict disease incidence in healthy eyes and disease progression in eyes with mild disease. That work must be done in a future longitudinal study. Third, it is possible that participants in the healthy cohort had subclinical keratoconus, which could confound the differences found between devices. Fourth, a second-generation (G2) ORA was not included in the study design. It would be expected that the G2 ORA would perform in a similar manner to the G1 ORA, given that they share working distances and air-puff-delivery and optical-detection systems. However, this hypothesis cannot be confirmed in the current study. Finally, we did not exclude participants who wore contact lenses, nor were contact lens wearers without their lenses for a set duration before the start of the study. Exclusion of contact lens wearers was not feasible for this study because rigid gas permeable lenses are the leading refractive error correction for patients with keratoconus (33, 34). Moreover, these lenses likely have little effect on the biomechanical parameters of the eye (35). Soft contact lenses may elicit corneal swelling (36), but they have not been shown to induce consistent changes to the biomechanical parameters of the ORA. Conflicting studies suggest that the use of soft contact lenses has no effect on CH (37), increases CH (38), or decreases CH (39). Such volatile results suggest that soft contact lenses did not have a meaningful effect on our results.

## 5 Conclusion

The changes made to the working distance and the air-puff-delivery and optical-detection systems of the G3 ORA altered its pressure-applanation waveforms and thus its biomechanical outcome metrics, compared to the G1 ORA. Additionally, there was meaningful variation between the IOP outcomes generated by the two devices. Thus, the G1 ORA and G3 ORA are not interchangeable devices. Clinicians should note the generation of the device used and then use the same generation for serial measurements over time. Researchers should report the generation of the device used to collect data in scientific communications. Caution must be taken when comparing results from a G1 ORA to those from a G3 ORA. Finally, both generations of the ORA can use KMI to differentiate eyes with keratoconus from healthy eyes, but future studies should update the KMI to maximize the diagnostic potential on the G3 device. Machine learning applications may be particularly well suited to aid in this task.

## Data Availability

The raw data supporting the conclusions of this article will be made available by the authors, without undue reservation.
