# Supplementary material for: Intraocular pressure and ocular biomechanical parameters vary between generations of the ocular response analyzer in healthy and ectatic eyes
Source: Front Med (Lausanne). 2025 Jul 24;12:1605641. doi: 10.3389/fmed.2025.1605641 (PMC12328285; doi:10.3389/fmed.2025.1605641)
Supplement: Supplementary file 1 [file Data_Sheet_1.docx]

Supplementary Material

# Supplementary Figures

Figure S1. Agreement between the Ocular Response Analyzer (ORA) G1 and ORA G3 for (A) waveform score (WFS), (B) keratoconus match index (KMI), (C) p1 area, (D) p2area, (E) w1, (F) w2, (G) h1, and (H) h2 in the healthy cohort. The center solid blue lines are inter-device mean difference, and the flanking dashed blue lines are the limits of agreement, from 1.96 to -1.96 standard deviations.

A





B





C





D





E





F





G





H





Figure S2. Agreement between the Ocular Response Analyzer (ORA) G1 and ORA G3 for (A) waveform score (WFS), (B) keratoconus match index (KMI), (C) p1 area, (D) p2area, (E) w1, (F) w2, (G) h1, and (H) h2 in the keratoconus cohort. The center solid blue lines are inter-device mean difference, and the flanking dashed blue lines are the limits of agreement, from 1.96 to -1.96 standard deviations.

A





B





C





D





E





F





G





H
